# Anlotinib plus icotinib as a potential treatment option for EGFR-mutated advanced non-squamous non-small cell lung cancer with concurrent mutations: final analysis of the prospective phase 2, multicenter ALTER-L004 study

**DOI:** 10.1186/s12943-023-01823-w

**Published:** 2023-08-05

**Authors:** Linlin Zhang, Liuchun Wang, Jingya Wang, Jinliang Chen, Zhaoting Meng, Zhujun Liu, Xiangli Jiang, Xinyue Wang, Chun Huang, Peng Chen, Yan Liang, Richeng Jiang, Jing Wang, Diansheng Zhong, Yanhong Shang, Yan Zhang, Cuiying Zhang, Dingzhi Huang

**Affiliations:** 1 Department of Medical Oncology, Tianjin Medical University General Hospital, No.154, Anshandao, Heping District, Tianjin, 300052 China; 2 Department of Thoracic Oncology, Tianjin Lung Cancer Center, Key Laboratory of Cancer Prevention and Therapy, Tianjin’s Clinical Research Center for Cancer, Tianjin Medical University Cancer Institute & Hospital, National Clinical Research Center for Cancer, Tianjin Cancer Institute & Hospital, Tianjin Medical University, Tianjin, 300060 P. R. China; 3 Hebei Key Laboratory of Cancer Radiotherapy and Chemotherapy, Department of Medical Oncology, Affiliated Hospital of Hebei University, Baoding, China; 4 Department of Oncology IV, First Hospital of Shijiazhuang, Shijiazhuang, China; 5 Cancer center, Inner Mongolia Autonomous Region People’s Hospital, Huhhot, People’s Republic of China

**Keywords:** Non-small-cell lung cancer, ErbB receptors, Prognosis, Safety, Anlotinib, Icotinib

## Abstract

**Background:**

Non-small cell lung cancer (NSCLC) patients with epidermal growth factor receptor (EGFR) mutation and concurrent mutations have a poor prognosis. This study aimed to examine anlotinib plus icotinib as a first-line treatment option for advanced NSCLC carrying *EGFR* mutation with or without concurrent mutations.

**Methods:**

This phase 2, single-arm, multicenter trial (ClinicalTrials.gov NCT03736837) was performed at five hospitals in China from December 2018 to November 2020. Non-squamous NSCLC cases with EGFR-sensitizing mutations were treated with anlotinib and icotinib. The primary endpoint was progression-free survival (PFS). Secondary endpoints included the objective response rate (ORR), disease control rate (DCR), overall survival (OS), and toxicity.

**Results:**

Sixty participants were enrolled, including 31 (52%) and 29 (48%) with concurrent mutations and pathogenic concurrent mutations, respectively. The median follow-up was 26.9 (range, 15.0-38.9) months. ORR and DCR were 68.5% and 98.2%, respectively. Median PFS was 15.1 (95%CI: 12.6–17.6) months which met the primary endpoint, median DoR was 13.5 (95%CI: 10.0-17.1) months, and median OS was 30.0 (95%CI: 25.5–34.5) months. Median PFS and OS in patients with pathogenic concurrent mutations were 15.6 (95%CI: 12.5–18.7) months and not reached (95%CI: 17.46 months to not reached), respectively. All patients experienced TRAEs, including 26 (43%) and 1 (1.7%) who had grade ≥ 3 and serious treatment-related adverse events (TRAEs).

**Conclusions:**

Anlotinib combined with icotinib was effective and well-tolerated as a first-line treatment option for EGFR mutation-positive advanced NSCLC with or without concurrent mutations.

**Trial registration:**

ClinicalTrials.gov identifier: NCT03736837.

## Background

Non-small cell lung cancer (NSCLC), the leading cause of death (85-90%) among all malignant lung tumors, is generally related to smoking and more prominent in individuals with advanced age (65-year-old or above). Several oncogenic driver mutations, e.g., epidermal growth factor receptor (EGFR; 15–20% of NSCLCs) and anaplastic lymphoma kinase (ALK; 5% of NSCLCs), have been shown to promote cell transformation and cancer growth and progression [1]. NSCLC patients harboring EGFR-sensitizing mutations display promising objective response to EGFR tyrosine kinase inhibitors (TKIs) [2–5]. EGFR-sensitizing mutations are the gold-standard biomarkers for predicting the suitability of first-line EGFR-TKI therapy in NSCLC patients. However, the benefits of these regimens might vary by mutation profile; recently development of next-generation sequencing (NGS) technologies allows detection of a large number of concurrent mutations in patients with EGFR-mutant NSCLC that further complicate the situation [6]. In recent studies [7–9], the existence of concurrent mutations substantially reduced progression-free survival (PFS) in patients administered EGFR TKI treatment.

Although osimertinib, a third-generation EGFR TKI, has superior efficacy as the first-line treatment based on the FLAURA study, its efficacy against concurrent mutations is still unclear. Recently, the LC-SCRUM study showed that the median PFS of osimertinib for EGFR-mutant NSCLC with concomitant amplification of RTK-related genes and cell cycle genes was approximately 8 months shorter than that of the FLAURA study [10]. Hence, the clinical therapeutic efficacy in NSCLC patients with EGFR-sensitizing mutations and concurrent mutations is unsatisfying, and new treatment modalities are needed for these patients.

The molecular mechanism of vascular endothelial growth factor (VEGF) inhibitors combined with EGFR inhibitors in the treatment of NSCLC suggests synergistic anti-tumor effects and drug resistance alleviation. In recently reported large-scale randomized studies including JO25567 [11], NEJ026[12], and RELAY [13–15], the addition of anti-angiogenic drugs to EGFR TKIs substantially improved PFS in TKI-naive patients with EGFR-mutant NSCLC. Patients with concomitant TP53 mutation showed particular benefits from the dual inhibition of EGFR and angiogenesis in the RELAY and ACTIVE trials [14–16]. However, the anti-angiogenesis monoclonal antibody required intravenous administration, which is less convenient than orally available dugs.

Anlotinib selectively suppresses VEGFR1/2/3, FGFR1/2/3 and PDGFRα/β, and is the first and only effective single-agent vascular-targeted drug for advanced NSCLC [17]. It is currently approved in China in refractory advanced NSCLC patients after ≥ 2 lines of systemic therapy [18] based on the ALTER0303 trial, in which anlotinib(12 mg once per day, 2 weeks on-treatment followed by 1 week off-treatment) prolonged PFS by 4 months and overall survival by 3.3 months compared with the placebo [19]. Hence, the combination of anlotinib plus EGFR TKIs could be potentially more effective and convenient than previously reported combination regimens.

Here, we report the efficacy and safety of a prospective multicenter trial evaluating a new combination regimen, anlotinib plus icotinib, as the first-line treatment of EGFR mutation-positive advanced NSCLC. More importantly, we evaluated the relationship between efficacy and genetic profile of this new regimen in the treatment of patients with EGFR-sensitizing mutations harboring concurrent mutations, in order to identify the population that would most benefit from this combination regimen.

## Methods

### Study design and patients

This phase2, single-arm, multicentre trial (ClinicalTrials.gov NCT03736837) was conducted at five research centers in China between December 2018 and November 2020. This study was approved by the independent institutional review boards or independent ethics committees associated with each participating center. All patients provided written informed consent before enrollment. Eligible patients had histologically or cytologically confirmed stage IIIB/IV or postoperative recurrent non-squamous NSCLC with EGFR-sensitizing mutations (exon 19 deletion or Leu858Arg mutation). Tumor samples were screened for EGFR mutations by NGS certified by CLIA or CAP. Patients aged 18–75 years with one or more measurable lesion(s) based on Response Evaluation Criteria in Solid Tumors (RECIST) 1.1. No previous chemotherapy or EGFR-TKIs for advanced disease was allowed, including neoadjuvant or adjuvant chemotherapy in the previous 6 months from the final administration date. Other inclusion criteria included Eastern Cooperative Oncology Group performance status 0 or 1; adequate hematological, hepatic, and renal functions; asymptomatic or mildly symptomatic brain metastases; and life expectancy ≥ 3 months at the time of enrollment.

Major exclusion criteria included confirmation of history or presence of hemoptysis or bloody sputum, any coagulation disorder, severe or uncontrolled hypertension, or tumors invading or abutting major blood vessels.

### Definition of concurrent mutations and pathogenic concurrent mutations

Concurrent mutations were defined as gene mutations detected by NGS besides EGFR exon 19 deletion or exon 21 L858R mutation but excluding the concurrent rare EGFR mutations in line with previous studies. Pathogenic concurrent mutations were defined as gene mutations besides EGFR exon 19 deletion or exon 21 L858R mutation, which was predicted as deleterious or potentially deleterious with at least two software among PolyPhen2, PROVEAN, and Mutation Taster.

### Treatment and follow-up

Patients were administered oral icotinib (125 mg three times per day) and anlotinib (12 mg once per day). Each cycle of anlotinib treatment was defined as 2 weeks on-treatment followed by 1 week off-treatment. Treatment was continued until disease progression, intolerable toxicity, or patient request of discontinuation. Dose adjustments were judged by the investigators based on treatment-related adverse events (TRAEs) following the NCI-CTCAE v4.0 criteria. Dose modifications (10 mg/d or 8 mg/d) of anlotinib were allowed according to the protocol-defined dose modification criteria. In case of dose reduction, the patient was not allowed to return to the previous dose level; if the dose of 8 mg/d was not tolerated, the treatment was permanently terminated.

Tumor response was evaluated every 6 weeks based on RECIST 1.1. Patient follow-up was performed to assess clinical outcomes, including toxicity, efficacy, and survival, until death. Follow-up for survival was performed via clinical visits or telephone calls every 12 weeks.

### Endpoints

The primary endpoint was PFS evaluated by investigators, defined as the time elapsed from patient enrollment to the first disease progression or death from any cause, whichever occurred first. Secondary endpoints included the objective response rate (ORR), disease control rate (DCR), and OS. ORR was defined as the proportion of patients achieving a complete (CR) or partial response (PR). DCR was defined as the proportion of participants who achieved CR, PR, and stable disease (SD). OS was defined as the time elapsed from patient enrollment to death from any cause or censored at the data cutoff date. Adverse events (AEs) were recorded and graded according to the National Cancer Institute Common Terminology Criteria for Adverse Events, version 4.0.

### NGS-based mutation profiling

Three prediction software (Mutation Taster, PolyPhen-2 and PROVEAN) [20, 21] were used to classify the detected mutations as pathogenic and non-pathogenic, using bioinformatics and programming to analyze the impacts of different mutations in EGFR sensitive patients on the efficacy of TKIs. PolyPhen-2, a software tool developed by Harvard University, uses direct physical and evolutionary comparative considerations to predict the likely impacts of amino acid substitutions on human protein structure and function [22]. Based on the final score reflecting the hazard of missense mutations, a cutoff value of 0.800 was used, meaning that missense mutations with a score above 0.8 are pathogenic [23]. Mutation Taster evaluates the pathogenic potential of mutations by analyzing DNA sequence alterations [24, 25]. The PROVEAN software, on the other hand, examines the effects of amino acid substitutions or insertional deletions on the biological functions of proteins, which in turn affects the malignant biological behavior of tumors [26]. In this study, at least two predictive software-defined deleterious mutations were considered pathogenic.

### TP53 destructive/non-destructive mutations

Previous clinical studies have focused on the impact of TP53 mutations within a certain exon on EGFR TKI efficacy and the results have been inconsistent [27–29]. This may suggest that the number of exons that harbors the mutations is not a reliable predictor for the impact on TKI efficacy. In some studies, TP53 mutations have been classified into destructive and non-destructive types [30, 31], which can indicate whether specific TP53 mutants still carry the function. Therefore, we introduced the concept of TP53 disruptive/non-disruptive mutations to explore the effect of TP53 mutations on TKI efficacy. Destructive mutations include: (i) any mutation that produces a stop codon (including nonsense, frameshift, and intron mutations); (ii) missense mutations located within the L2 or L3 loop replacing one residue by another of different polarity or charge; (iii) in-frame deletions within the L2 or L3 loop. Non-destructive mutations are all mutations not categorized as destructive and include: (i) missense mutations and in-frame deletions located outside the L2-L3 loop; (ii) missense mutations within the L2-L3 loop but replacing one residue with another of the same polarity or charge.

### Statistical analysis

Referring to previous studies and data for similar agents, the median PFS of icotinib in the first-line treatment of EGFR-positive NSCLC was estimated at 9.9 months [32]. Combining the ALTER0302 trial, ALTER0303 trial, and current clinical practice, it was assumed the PFS of anlotinib combined with icotinib in the first-line treatment of EGFR-positive NSCLC would be 15 months. The single-sample one-sided Z-test was selected to yield 80% power at the α = 0.05 level, and the log-rank test was used for sample size correction using PASS 15.0. Assuming a 10% dropout rate, a sample size of at least 58 patients was required.

The full analysis set (FAS), based on the intention-to-treat (ITT) population, included all participants who used the study drugs at least once. EAS(efficacy analysis set)was used for efficacy analysis, which at least first response evaluation. The safety set (SS) included all participants who used the study drugs at least once with available safety assessments after using the study drugs. SS was used for safety analysis.

Statistical analysis was performed using SAS 9.1.3 (SAS Institute, Cary, NC, USA). Continuous data were presented as mean ± standard deviation if normally distributed or median (minimum, maximum) if skewedly distributed. Categorical data were described as n (%), and 95% confidence intervals (CIs) were determined. The Kaplan-Meier method was utilized to display PFS and OS, and safety analysis was mostly descriptive statistical analysis. Survival curves were compared by the log-rank test. P < 0.05 indicated statistical significance.

## Results

### Patients

From December 2018 to November 2020, totally 60 patients were enrolled in this study; all of them received anlotinib plus icotinib at least once and were included in the SS and FAS (Table 1). Median patient age was 62 years (range, 35–72) and 34 (43%) patients were male. The clinical stage was IV in 58 (98%) patients. Totally 30 (50%) cases had the EGFR 19del mutation, 30 (50%) had the L858R mutation, 31 (52%) had concurrent mutations, and 29 (48%) had pathogenic concurrent mutations. Two patients withdrew consent before the first efficacy evaluation, and the clinical efficacy for one patient was not assessed due to AEs; the remaining 57 patients were included in the EAS.

**Table 1:** Baseline characteristics of the participants

Characteristic	ITT (n = 60)	ESA (n = 57)
Median Age (range), years	62 (35–72)	62 (35–72)
≥60 years	35 (58.3%)	35(59.6%)
Sex (male)	26 (43.3%)	25(43.9%)
Clinical stage		
IIIB	1 (1.7%)	1(1.8%)
IIIC	1 (1.7%)	1(1.8%)
IV	58 (96.7%)	55(96.5%)
Smoking history		
Ever	11 (18.3%)	11(19.3%)
Current	9 (15.0%)	7(12.3%)
Never	40 (66.7%)	39(68.4%)
ECOG PS		
0	22 (36.7%)	22(38.6%)
1	37 (61.7%)	34(59.6%)
2	1 (1.7%)	1(1.8%)
Recurrent NSCLC	2 (3.3%)	2(3.5%)
Brain metastases	21 (35.0%)	21(36.8%)
*EGFR* mutations		
19del	30 (50.0%)	30(52.6%)
L858R	30 (50.0%)	27(47.4%)
Concurrent mutations	31 (51.7%)	30(52.6%)
Pathogenic concurrent mutations	29 (48.3%)	28(49.1%)

Data are presented as median (range) or n (%).

ITT, intention-to-treat; EAS, efficacy analysis set; ECOG PS, Eastern Cooperative Oncology Group performance status; NSCLC, non-small-cell lung cancer; EGFR, epidermal growth factor receptor gene.

### Efficacy

**Fig. 1:**
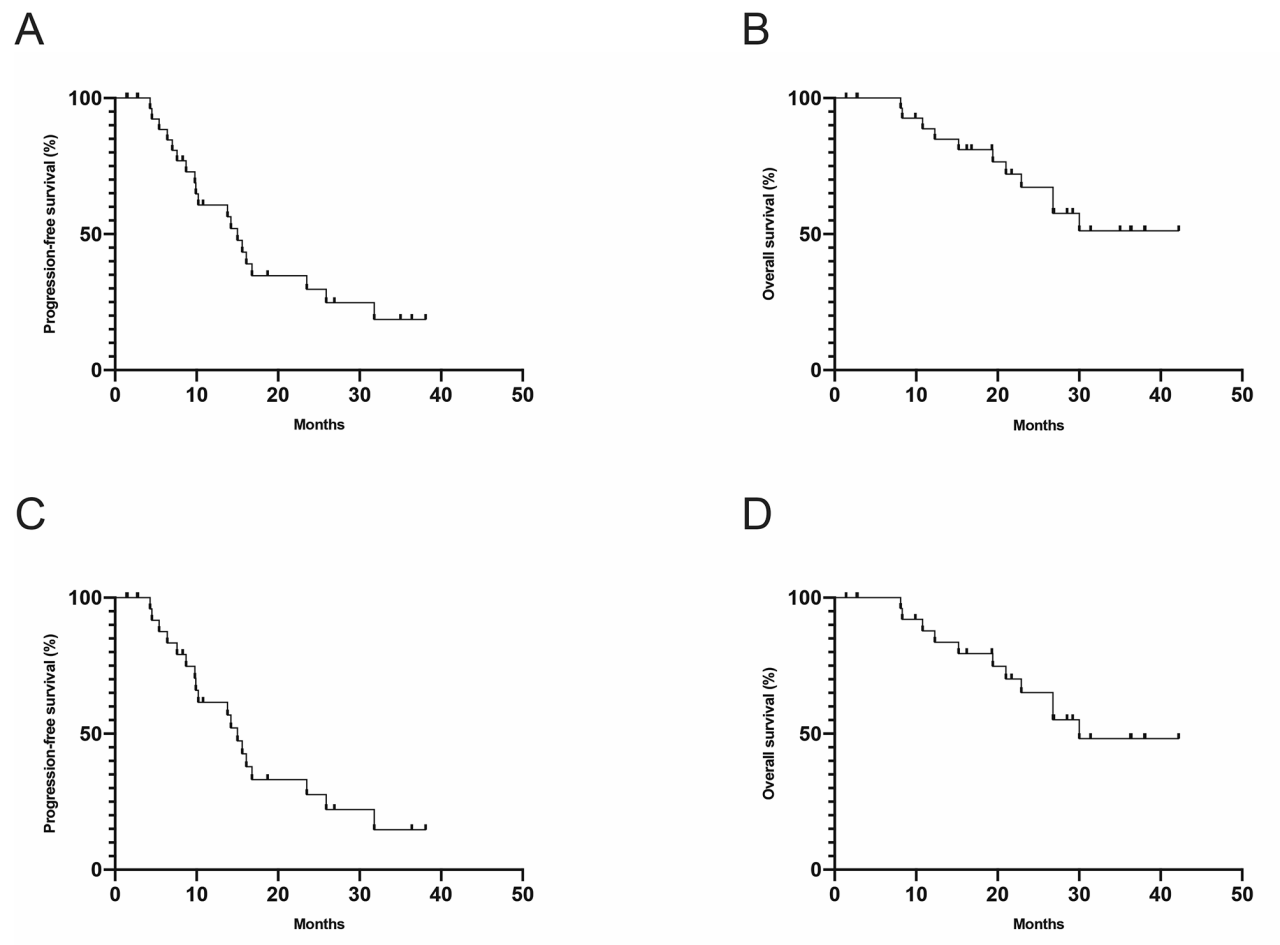
Efficacy of the combination of anlotinib + icotinib. Kaplan-Meier curves of PFS and OS in the ITT population **(A, B)** and cases with pathogenic concurrent mutations **(C, D)**

**Fig. 2:**
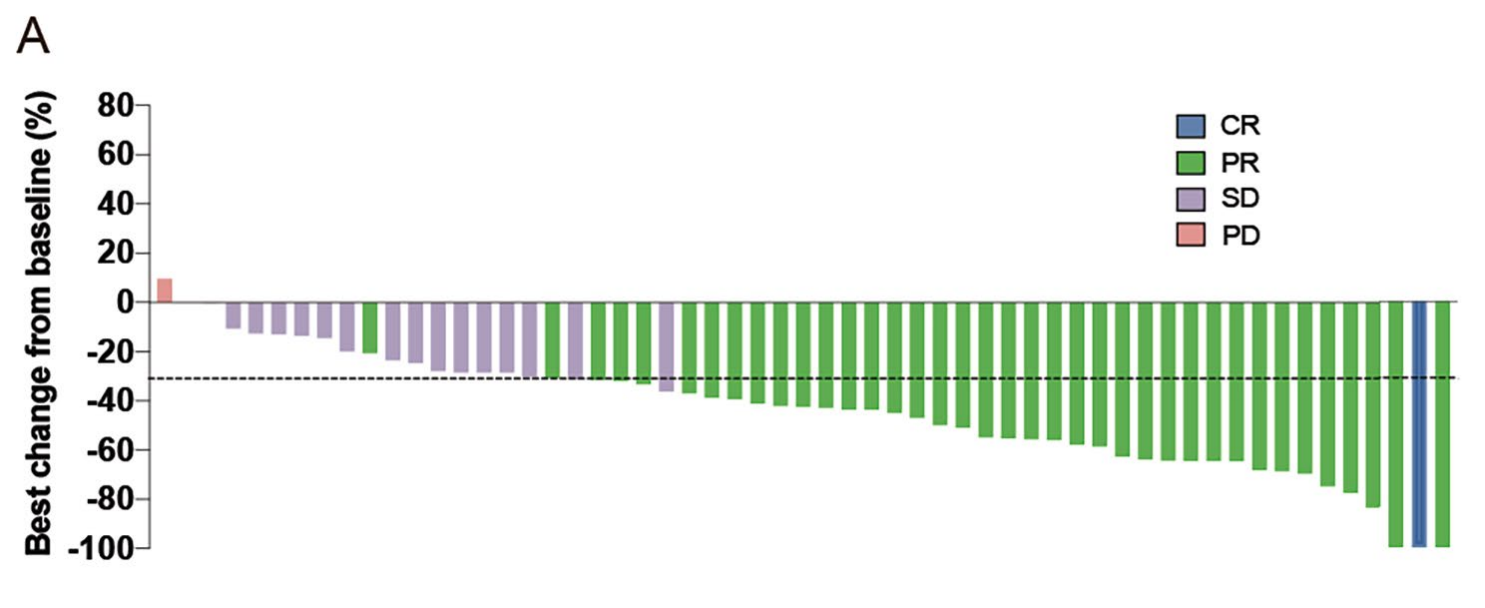
Best percentage changes from baseline in target lesion size in the efficacy analysis set. CR, complete response; PR, partial response; SD, stable disease; PD, disease progression

**Fig. 3:**
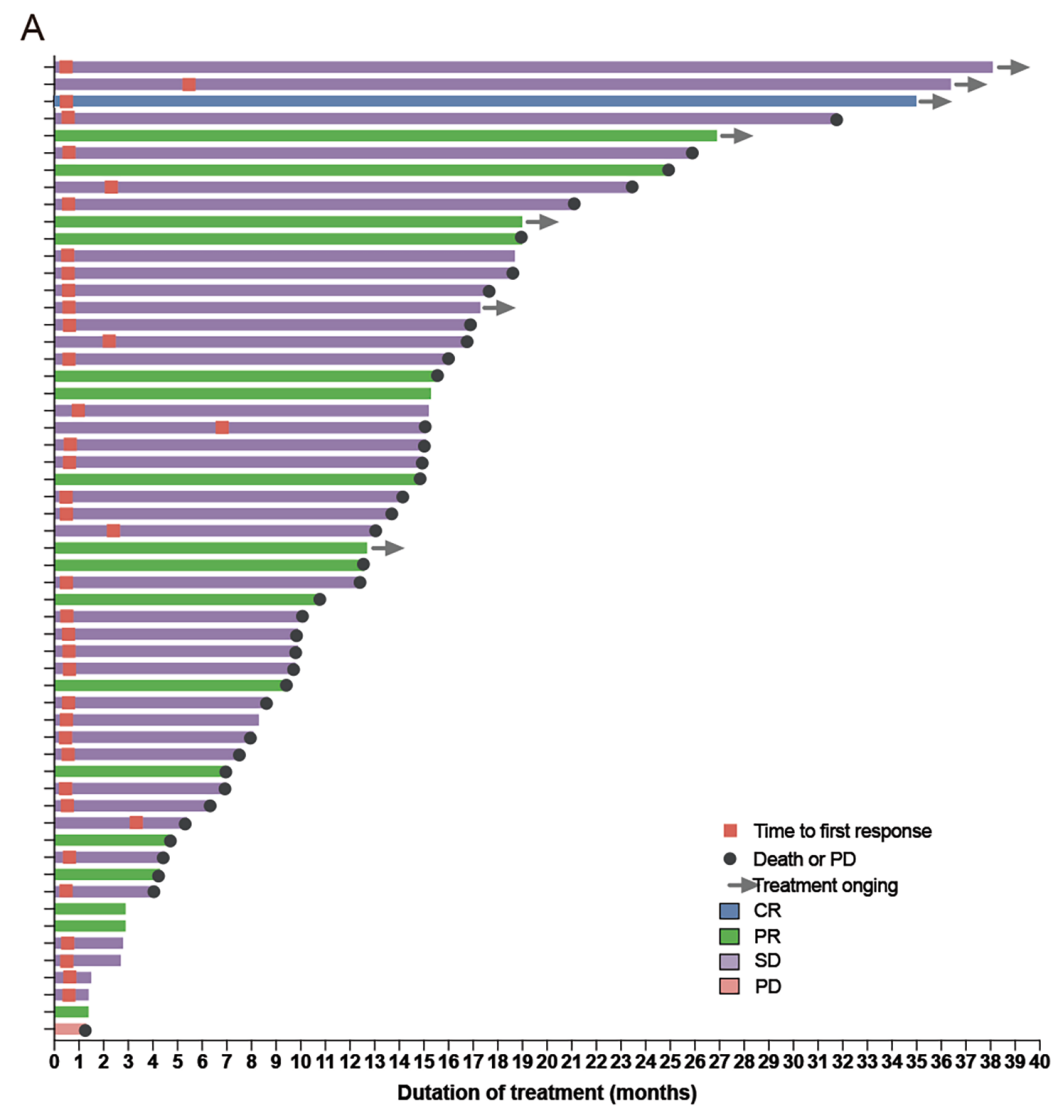
Treatment response and duration of response

The results of this study have met the statistical hypothesis (PFS = 15mons). As of October 26, 2022, the median follow-up time was 26.9 (range, 15.0-38.9) months. Median PFS and OS were 15.1 (95%CI: 12.6–17.6) and 30.0 (95%CI: 25.5–34.5) months, respectively (Fig. 1A and B). In the Per Protocol Set (PPS), the ORR was 68.5% (39/57), and one patient achieved CR. The DCR was 98.2% (56/57) (Fig. 2). Treatment response and duration of response (DoR) are presented in Fig. 3. The median DoR was 13.5 (95%CI: 10.0-17.1) months. As of the data cutoff date, 7 patients were still receiving treatment, 22 were deceased, 38 had discontinued treatment due to PD, and 6 had discontinued treatment due to AEs. Median PFS and OS in patients with pathogenic concurrent mutations were 15.6 (95%CI: 12.5–18.7) months and not reached (95%CI: 17.46 months to not estimable), respectively (Fig. 1C and D). With subgroup analyses, there were no differences in median PFS according to the co-mutation status (P = 0.623), pathogenic co-mutation status (P = 0.885), EGFR mutation type (P = 0.214), brain metastasis (P = 0.417), liver metastasis (P = 0.428), bone metastasis (P = 0.334), dose interruption (P = 0.227), dose reduction (P = 0.970), and grade 3/4 AEs (P = 0.464).

### Mutation frequencies and concurrent mutations

**Fig. 4:**
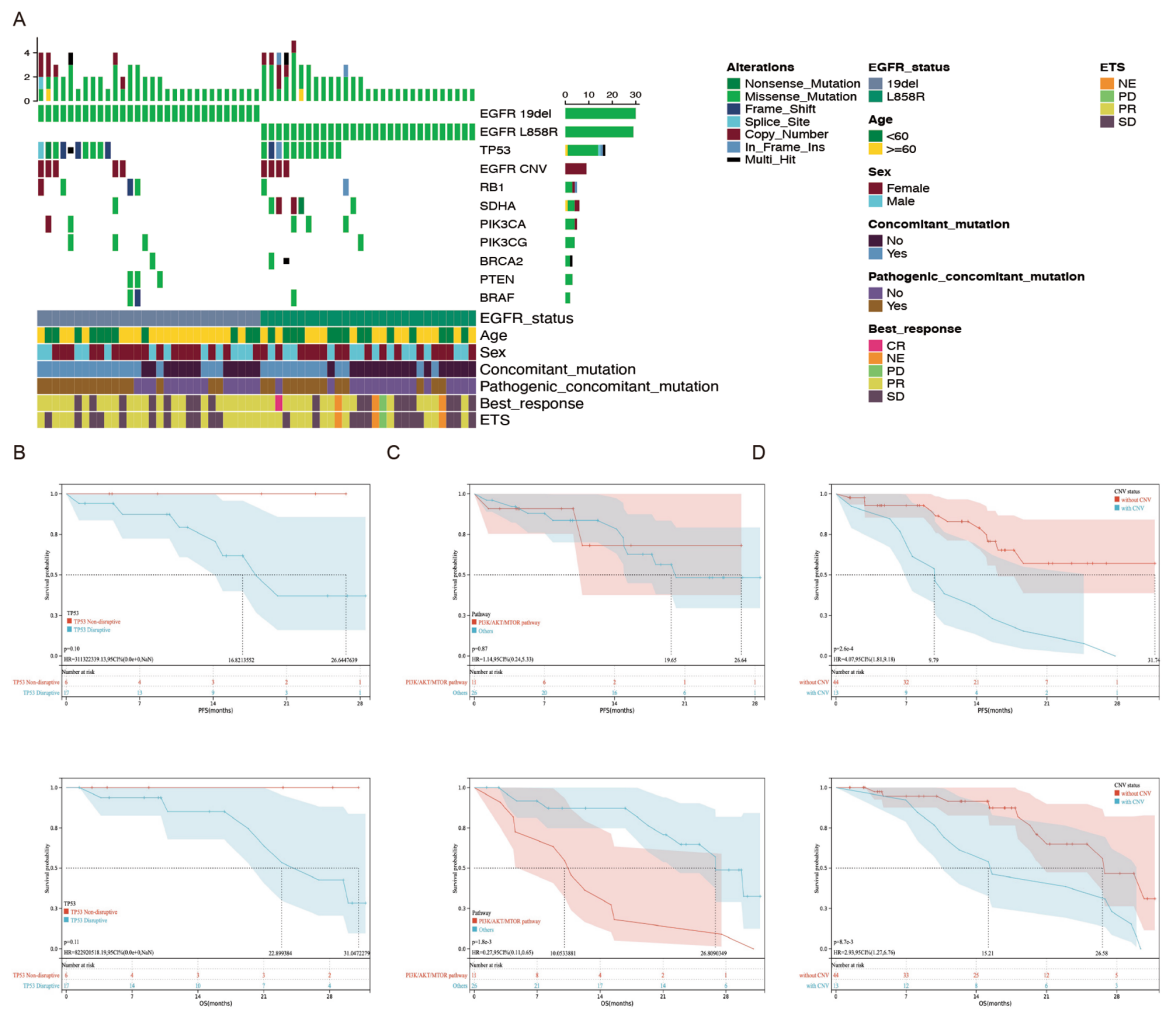
Mutation frequencies and concurrent mutations **(A)** Landscape of concurrent mutations in the intention-to-treat population. **(B)** Progression-free survival (PFS) and overall survival (OS) in patients with disruptive and non-disruptive TP53 mutation. **(C)** PFS and OS in patients with concurrent mutations located in PI3K/AKT/mTOR or other pathways. **(D)** PFS and OS in patients with or without copy number variation (CNV).

ORR was remarkably different between cases with and without pathogenic concurrent mutations (83% vs. 43%), and DCR was 100% vs. 93%. The baseline landscape of concurrent mutations in 60 patients obtained by NGS of tissue samples is shown in Fig. 4A. Of the 37 patients, 23 had TP53 mutations (one of whom had multiple TP53 mutations). Non-destructive TP53 mutations were present in 6 of the 23 patients. Survival data are shown in Fig. 4B. Although there were no statistically significant differences in PFS and OS because of the limited sample size, patients with non-disruptive TP53 mutations had numerically longer PFS and OS. Of the 37 patients, 11 with concurrent mutations located in the PI3K/AKT/MTOR pathway had significantly shorter OS (P = 0.0018) (Fig. 4C). The baseline copy number variation (CNV) landscape was comparable among the patients. Among the 37 patients with concurrent mutations, 13 had CNV. Patients with CNV had significantly shorter PFS (P = 0.0003) and OS (P = 0.0087) under combination treatment (Fig. 4D).

### Safety

All patients experienced TRAEs, among whom 26 (43.7%) had grade ≥ 3 TRAEs and 1 (1.7%) had a serious TRAE (Table 2). TRAEs led to dose interruption of any drug, dose reduction of anlotinib, and treatment discontinuation in 23 (38.3%), 15 (25.0%), and 5 (8.3%) patients, respectively. These TRAEs included hypertriglyceridemia, proteinuria, hypertension, hypercholesterolemia, hand-foot syndrome and positive fecal occult blood. There was no occurrence of ILD in this study. The most common all-grade TRAEs (frequency of ≥ 15%) were hypertriglyceridemia (65%), hypertension (57%), hypercholesterolemia (52%), proteinuria (50%), diarrhea (50%), hand-foot syndrome (35%), hypothyroidism (33%), elevated thyroid stimulating hormone (28%), rash (27%), elevated alanine transaminase (25%), elevated low-density lipoprotein (22%), elevated aspartate aminotransferase (20%), fecal occult blood (17%), bleeding gums (17%), oral mucositis (15%), urine occult blood (15%), and hematuria (15%). The most common grade ≥ 3 TRAEs (frequency of > 5%) were hypertension (25%) and diarrhea (5%). The only grade 4 TRAE was hypertriglyceridemia (n = 1, 2%) (Table 3).

**Table 2:** Treatment-related adverse events

Events	Safety set (n = 60)
Any TRAE	60 (100.0%)
Grade ≥ 3 TRAEs	26 (43.3%)
Serious TRAEs	1 (1.7%)
TRAEs leading to dose interruption, any drug	23 (38.3%)
TRAEs leading to dose interruption, anlotinib	23 (38.3%)
TRAEs leading to dose interruption, icotinib	2 (3.3%)
TRAEs leading to dose reduction, any drug	15 (25.0%)
TRAEs leading to dose reduction, anlotinib	15 (25.0%)
TRAEs leading to dose reduction, icotinib	0
Discontinued combined therapy due to TRAEs	5 (8.3%)

TRAE, treatment-related adverse event.

**Table 3:** Preferred terms of the treatment-related adverse events (TRAEs)

TRAE	Grade 1/2	Grade 3	Grade 4	Total
Hypertriglyceridemia	37 (62%)	1 (2%)	1 (2%)	39 (65%)
Hypertension	19 (32%)	15 (25%)	0 (0%)	34 (57%)
Hypercholesterolemia	31 (52%)	0 (0%)	0 (0%)	31 (52%)
Proteinuria	30 (50%)	0 (0%)	0 (0%)	30 (50%)
Diarrhea	27 (45%)	3 (5%)	0 (0%)	30 (50%)
Hand-foot syndrome	19 (32%)	2 (3%)	0 (0%)	21 (35%)
Hypothyroidism	20 (33%)	0 (0%)	0 (0%)	20 (33%)
Elevated thyroid stimulating hormone	17 (28%)	0 (0%)	0 (0%)	17 (28%)
Rash	16 (27%)	0 (0%)	0 (0%)	16 (27%)
Elevated alanine transaminase	13 (22%)	2 (3%)	0 (0%)	15 (25%)
Elevated low-density lipoprotein	13 (22%)	0 (0%)	0 (0%)	13 (22%)
Elevated aspartate aminotransferase	11 (18%)	1 (2%)	0 (0%)	12 (20%)
Fecal occult blood	10 (17%)	0 (0%)	0 (0%)	10 (17%)
Bleeding gums	9 (15%)	1 (2%)	0 (0%)	10 (17%)
Oral mucositis	8 (13%)	1 (2%)	0 (0%)	9 (15%)
Urine occult blood	9 (15%)	0 (0%)	0 (0%)	9 (15%)
Hematuria	9 (15%)	0 (0%)	0 (0%)	9 (15%)
Nasal bleeding	8 (13%)	0 (0%)	0 (0%)	8 (13%)
Hyperuricemia	6 (10%)	0 (0%)	0 (0%)	6 (10%)
Sinus bradycardia	2 (3%)	1 (2%)	0 (0%)	3 (5%)
Thrombocytopenia	3 (5%)	0 (0%)	0 (0%)	3 (5%)
Hyperbilirubinemia	1 (2%)	0 (0%)	0 (0%)	1 (2%)
Hemoptysis	1 (2%)	0 (0%)	0 (0%)	1 (2%)
Increased creatinine	1 (2%)	0 (0%)	0 (0%)	1 (2%)
Acute coronary syndrome	0 (0%)	1 (2%)	0 (0%)	1 (2%)
Intracranial hypertension	0 (0%)	1 (2%)	0 (0%)	1 (2%)
Myocardial infarction	0 (0%)	1 (2%)	0 (0%)	1 (2%)
Thromboembolic events	0 (0%)	1 (2%)	0 (0%)	1 (2%)

## Discussion

EGFR TKIs are recommended as the standard first-line treatment for NSCLC with EGFR-sensitizing mutations with the third-generation EGFR TKIs being the preferred agents nowadays. Nevertheless, the FLAURA Asian subgroup analysis revealed no statistically significant OS benefit with the curve crossing at approximately 39 months (hazard ratio [HR] = 1.00, 95%CI: 0.75–1.32) [33]. Similar results were reported in the FLAURA China extension cohort (HR = 0.848, 95%CI: 0.557–1.291) [34]. The ALTER-L004 trial was a multicenter, phase 2 single-arm exploratory clinical trial strongly suggesting the efficacy and safety of anlotinib combined with icotinib in patients with EGFR-mutated advanced NSCLC. To the best of our knowledge, this study was comprehensive analysis of the associations of concurrent mutations with response to the dual inhibition of angiogenesis and EGFR in the first time.

Median PFS in this trial was 15.1 months, corroborating other studies of EGFR-TKIs combined with anti-angiogenic drugs. Indeed, in a study by Zhang et al. [35], median PFS in the gefitinib plus anlotinib group was 11.53 months. The JO25567 phase II clinical trial showed median PFS in the erlotinib monotherapy and erlotinib plus bevacizumab groups of 9.7 and 16 months, respectively [11]. In the RELAY study [13–15], PFS was significantly longer in the ramucirumab plus erlotinib group compared with the placebo plus erlotinib group (median, 19.4 vs. 12.4 months), with a stratified HR of 0.59 (95%CI: 0.46–0.76; P < 0.0001); subgroup analyses revealed that TP53 co-mutation was associated with superior outcomes for RAM + ERL in both the ex19del and ex21L858R subgroups. This dual regimen of oral apatinib plus gefitinib provides a convenient first-line option for EGFR-mutant cases. In the phase 3 ACTIVE study [16], median PFS in the apatinib plus gefitinib and placebo plus gefitinib groups was 13.7 and 10.2 months (HR = 0.71, P = 0.0189), respectively. Post hoc analysis revealed that PFS benefits tended to favor the apatinib plus gefitinib group in patients with TP53 exon 8 mutation.

In a study by Zhang et al. [9], concomitant mutation with TKI-naive treatment was significantly associated with reduced ORR (44% vs. 77%; P = 0.01), shorter median PFS (6.20 vs. 18.77 months, P < 0.001), and shorter median OS (22.70 vs. not reached, P < 0.001). The BENEFIT study by Wang et al. [8] showed a median PFS of 13.2 months in patients with EGFR-sensitizing mutations only, versus 9.3 months in patients with EGFR-concomitant tumor-suppressor-gene mutations and only 4.6 months in those with EGFR-concomitant oncogene mutations.

This work showed that anlotinib plus icotinib could achieve promising ORR and PFS in patients with concomitant mutations. Although first line osimertinib may bring better PFS, patients harboring concurrent mutations such as TP53 still have shorter PFS than reported in the present trial [36–38]. This finding suggested single-pathway-blockade could not improve survival with single drug escalation remodeling, and combination therapy is required. Meanwhile, PFS of patients administered the combination of anti-angiogenesis and EGFR TKIs in the entire study population was not superior to that of the osimertinib group, suggesting that such a combination regimen requires a precise selection of the target population. It is possible that cases with combined concurrent mutations are more likely to achieve potential benefits. Improved ORR was observed in the co-mutation sub-group, probably owing to the bias caused by insufficient sample size. It is also possible that patients with concurrent mutations are more appropriate for the dual oral inhibition regimen.

Based on existing data, we recognize that the complexity of the tumor genome determines the treatment of tumors rather than single gene targeting. The occurrence of concurrent mutations is likely to completely change the biological properties of the original tumor through synergistic effects, conferring new biological features to the tumor and leading to drug resistance. These concurrent mutations are likely to occur gradually during the treatment process. Although existing studies have suggested a value for concurrent mutations, most of them did not statistically analyze the associations of specific mutation sites with tumor cell functions. In addition to some common mutations, mutations in non-hotspot genes may also play important roles in tumor development. Therefore, further investigation is urgently required to identify the regularity of the occurrence and development of concurrent mutations in NSCLC as well as their impacts on clinical prognosis. Predicting deleterious mutations is widely used in the era of precision therapy in cancer. In this study, we established gene mutation profiles through the NGS technology, used bioinformatics to predict the pathogenicity of non-hot-spot variants with unknown biological significance, searched for clinically relevant concurrent mutations, and examined the effects of concurrent mutations on the efficacy of TKIs and their relationships with drug resistance. For missense mutations in non-hotspot genes, we applied three recognized prediction software (MutationTaster, Polyphen-2 and PROVEAN), elucidating the pathogenic potential of the mutations through DNA sequencing and amino acid alterations. In the era of precision tumor therapy, these methods for predicting deleterious mutations have been widely used [39–43].

TP53, a widely studied and critical tumor suppressor in tumor development, also plays a significant role in the efficacy of EGFR TKI treatment. Previously, the effect of TP53 mutation within a certain exon (e.g., exon 8) on TKI has been studied but inconsistent conclusions were obtained. Therefore, the concept of disruptive/non-disruptive was introduced [18, 19]. Destructive mutations result in complete or almost complete loss of function of the p53 protein. In contrast, non-destructive mutations preserve some functional features of wild type p53, known as gain of function. Previous studies have shown that non-disruptive p53 mutations are associated with reduced patient survival in advanced NSCLC [44]. However, according to the present analysis, the anlotinib plus icotinib regimen had improved efficacy in cases harboring non-disruptive TP53 mutations (although statistical significance was not reached, probably due to insufficient sample size). To the best of our knowledge, this is the first and only clinical study proposing this notion, and large sample studies are warranted for validation.

In the present study, five patients discontinued the combined therapy due to TRAEs. No grade 5 AEs occurred, and no new safety signals were observed the present study. As to anlotinib, dose interruption and dose reduction due to TRAEs occurred in 23 and 15 patients, respectively, and two patients had a starting dose of 8 mg. The dosing of anlotinib combined with EGFR-TKIs deserves further investigation.

Major limitations of the present study included the limited sample size, the single-arm design, and the inconsistency of serial biopsies. Additionally, distinct detection platforms as well as panel sizes and depths may represent an underestimation of concomitant mutations. Compared to previous studies, survival and response rate in patients without concurrent mutations did not seem to increase in this study, likely due to the following reasons: (1) limited sample size; (2) false negatives due to limited sequencing depth and panel size; (3) the dual inhibition regimen may not be applicable to cases without concurrent mutations; (4) the first-generation TKI icotinib was applied in this study, whose efficacy is lower compared with a third generation TKI.

In conclusion, anlotinib combined with icotinib is effective and tolerable in the first-line treatment of EGFR mutated advanced NSCLC with or without concurrent mutations. These results call for further large-scale randomized controlled trials. According to the findings of this study, patients harboring pathogenic concurrent mutations are more suitable for the anlotinib + icotinib regimen option. Our team is now conducting a multicenter, phase III RCT clinical trial in EGFR-mutant NSCLC patients with pathogenic concurrent mutations, comparing anlotinib combined with icotinib vs. icotinib (NCT04797806). It is expected that further analysis of patients harboring concurrent mutations will be further studied with the aim of achieving better outcomes.

## Conclusions

In summary, dual inhibition of anlotinib + icotinib was effective and well-tolerated as a first-line treatment option for EGFR mutation-positive advanced NSCLC with or without concurrent mutations. Identifying concurrent mutations as prognostic factors in EGFR mutant NSCLC is crucial to patient stratification and selection of treatment strategies. Our findings lay the foundation for a strategy to stratify patients based on concurrent mutations. Moreover, TP53 with non-destructive mutations plays a significant role in the efficacy of EGFR TKI treatment.

## Data Availability

The data that support the findings of this study are available from the corresponding authors upon reasonable request. All requests for raw data will be reviewed by the Tianjin Medical University Cancer Institute & Hospital, Tianjin Medical University General Hospital, Affiliated Hospital of Hebei University, First Hospital of Shijiazhuang, and Inner Mongolia Autonomous Region People’s Hospital.

## Abbreviations

AEs: Adverse events
ALK: anaplastic lymphoma kinase
CI: confidence intervals
CNV: copy number variation
CR: complete response
DCR: disease control rate
DoR: duration of response
EAS: efficacy analysis set
EGFR: epidermal growth factor receptor
FAS: full analysis set
ITT: intention-to-treat
NGS: next-generation sequencing
NSCLC: non-small cell lung cancer
ORR: objective response rate
OS: overall survival
PFS: progression-free survival
PPS: Per Protocol Set
PR: partial response
SD: stable disease
SS: safety set
TKIs: tyrosine kinase inhibitors
TRAEs: treatment-related adverse events
VEGF: vascular endothelial growth factor
